# Is histologic esophagitis associated with dental erosion: a cross-sectional observational study?

**DOI:** 10.1186/s12903-017-0408-z

**Published:** 2017-08-10

**Authors:** Lynn Roosa Friesen, Brenda Bohaty, Robin Onikul, Mary P. Walker, Caren Abraham, Karen B. Williams, Jose T. Cocjin, Eileen L. Cocjin, Craig A. Friesen

**Affiliations:** 1 Department of Research and Graduate Programs, University of Missouri - Kansas City School of Dentistry, 650 E 25th Street, Room 101-O, Kansas City, MO 64108 USA; 2 Department of Pediatric Dentistry, University of Missouri – Kansas City School of Dentistry, Department of Dentistry – Children’s Mercy Kansas City, 2401 Gillham Road, Kansas City, MO 64108 USA; 3 Department of Dentistry, Children’s Mercy Kansas City, 2401 Gillham Road, Kansas City, MO 64108 USA; 4 Departments of Oral & Craniofacial Sciences and Restorative Clinical Sciences, University of Missouri - Kansas City School of Dentistry, 650 E 25th Street, Kansas City, MO 64108 USA; 5 University of Missouri – Kansas City School of Dentistry, 650 E 25th Street, Kansas City, MO 64108 USA; 6 Department of Biomedical and Health Informatics, University of Missouri – Kansas City School Medicine, 1000 E. 24th St, Kansas City, MO 64108 USA; 7 Division of Gastroenterology, Hepatology, and Nutrition – Children’s Mercy Kansas City, 2401 Gillham Road, Kansas City, MO 64108 USA; 8 Department of Pediatric Dentistry, University of Missouri – Kansas City School of Dentistry, 650 E 25th Street, Kansas City, MO 64108 USA

**Keywords:** Tooth erosion, GERD, Gastro-oesophageal reflux disease, Esophagitis

## Abstract

**Background:**

Gastroesophageal reflux disease (GERD) affects 15–25% of children and adolescents in the United States. The diagnosis of GERD in children is complex as reported symptoms or symptom profiles have been found to be unreliable. Frequently, the diagnosis must be confirmed by objective tests such as pH monitoring or histological evidence of esophagitis on an esophageal biopsy. Dental erosion has been shown to be associated with GERD as an atypical complication and has the potential to be a marker of GERD. The purposes of this study were to compare the frequency and patterns of dental erosion in children and adolescents with and without histologic esophagitis.

**Methods:**

Twenty-five subjects were recruited from patients scheduled for an upper gastrointestinal endoscopy. Information regarding potential GERD symptoms, food habits, and dental hygiene habits were obtained. Intra-oral photographs were taken, and a dental exam for erosion was performed. The results of a standard biopsy taken from the lower third of the esophagus during an endoscopy were used to divide subjects into either the control group or the GERD group (i.e. those with histologic esophagitis).

**Results:**

Twenty-two subjects yielded 586 evaluable teeth. No significant difference was found between frequency or erosion patterns of those with and without histologic esophagitis. Dental erosions were more frequent in primary teeth.

**Conclusions:**

Dental erosions do not appear to be associated with histologic esophagitis indicative of GERD.

**Electronic supplementary material:**

The online version of this article (doi:10.1186/s12903-017-0408-z) contains supplementary material, which is available to authorized users.

## Background

Gastroesophageal reflux (GER) is a common condition in infants and children [1]. When associated with complications, GER becomes gastroesophageal reflux disease or GERD. In addition to the well documented complications of GERD such as vomiting, hematemesis, respiratory disease, and poor growth, it may also play a role in the development of dental erosions [2]. Once dentin is exposed to acid, the loss of tooth structure accelerates. This problem may be particularly troublesome in primary teeth whose enamel and dentin are much thinner than permanent teeth [3].

**Fig. 1:**
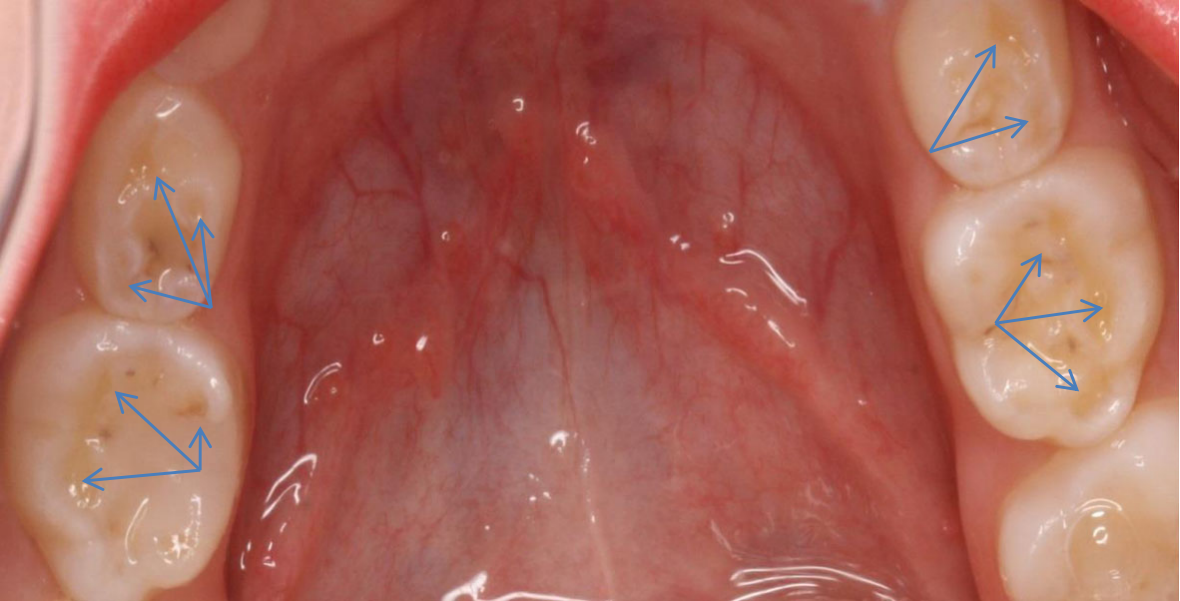
Cusp Tip Erosion Present on Primary Molars Displaying “Dished Out” Lesions

**Figure 2:**
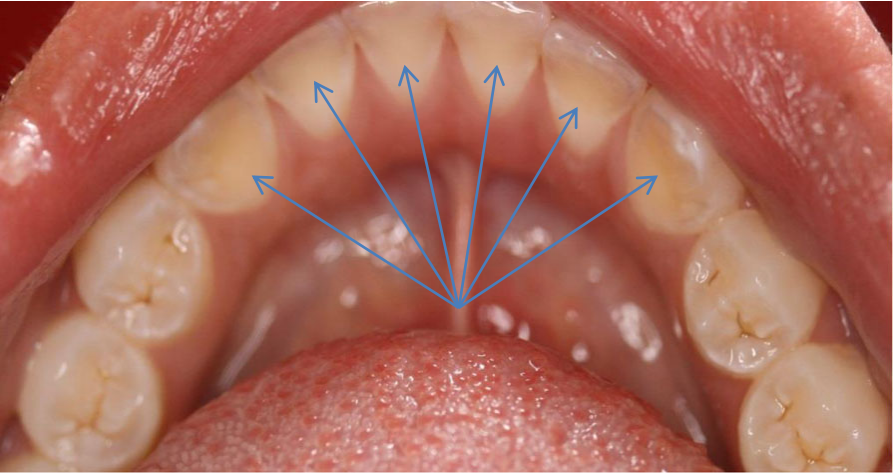
Erosion Present on the Lingual Surfaces of Permanent Mandibular Incisors and Cuspids

Epidemiological studies have shown that the prevalence of dental erosion in children varies widely between 2 and 57% [4]. Dental erosion, also known as perimyolysis, is the irreversible loss of dental hard tissue by a chemical process in the absence of bacteria. This differentiates it from caries. It is characterized as a hard “dished out” area with a smooth, glistening base (Fig. 1). Both extrinsic and intrinsic factors may contribute to erosion. Extrinsic factors include the consumption of acidic foods or beverages, medications taken in the form of syrup, chewable Vitamin C tablets, medications that cause xerostomia, inhalers used for asthma that alter the pH, and snacks [5, 6]. Intrinsic factors include eating disorders, chronic vomiting, persistent regurgitation and rumination, and gastroesophageal reflux. A multitude of factors may modify the erosion process, such as saliva volume and content, oral hygiene practices, and the presence or absence of fluoride, caries, enamel hypoplasia, and swishing and holding drinks in the mouth [4, 7, 8]. The pattern of damage depends on the etiology. Most extrinsic factors affect the facial surface of the maxillary anterior teeth, whereas intrinsic factors tend to cause damage to the lingual surfaces of the teeth [9] (Fig. 2).

Eight studies have previously evaluated the possible association between GERD and dental erosions in children and adolescents, with mixed findings [4, 10–16]. Of these, only five utilized a control group [4, 12–14, 16]. Studies have varied with regard to patient selection and especially with regard to how GERD was defined as well as how erosions were quantified, by tooth or by subject. None of the previous studies have had GERD confirmed with esophageal biopsy. Several of the previous studies made the diagnoses of GERD by symptoms, and the diagnosis of GERD by symptoms in children and adolescents is not reliable [1].

The aim of this study was to determine whether the rate of dental erosion differ between children/adolescents with GER-associated esophagitis and normal histology, respectively. Differing from previous studies, we sought to define both patients and controls by esophageal histologic criteria and to analyze teeth clustered within individuals.

## Methods

This was a cross-sectional, observational study performed at a single site. The protocol was submitted to the Pediatric IRB at Children’s Mercy Kansas City and received approval (IRB # 12110509). Each subject and/or subject’s guardian signed an informed consent or assent as appropriate, prior to the subject’s participation in the study. The privacy rights of the subjects were observed.

### Subjects

A convenience sample of 25 patients presenting for esophagogastroduodenoscopy (EGD) for upper gastrointestinal symptoms were recruited from Children’s Mercy Kansas City. The patients who are evaluated in the Gastroenterology Clinic include a population of referral patients with all types of gastrointestinal diseases. Patients are referred from urban, suburban and rural settings. The subjects in the study ranged in age from 3 to 17. Subjects were ineligible for participation if they had any significant conditions or chronic diseases requiring regular medical care, continuous use of acid-reducing medications for greater than 8 weeks prior to endoscopy, any use of steroids (oral or inhaled) in the 8 weeks prior to endoscopy, were currently in an orthodontic appliance, or had extensive dental restorations throughout the mouth that would obscure the ability to evaluate the natural tooth surfaces for erosion.

### Procedures

#### Gastrointestinal symptoms

Subjects and their parents completed a gastrointestinal symptoms questionnaire created for this study. The questionnaire assessed the presence of various symptoms and if present, the subjects rated severity on a 3-point Likert scale (mild, moderate, severe) and estimated episode frequency per week [17]. Symptoms included pain (in general and with consumption of fried and spicy foods), dysphagia, nausea, vomiting/regurgitation, chest pain, heartburn, excessive belching, bad breath, excessive crying, poor sleep, and relief from anti-acid medications. In order to capture extent of symptom severity, a composite score was computed as a product of symptom severity (mild = 1, moderate =2 and severe =3) and number of episodes per week. The gastrointestinal symptoms questionnaire is located in the “Additional files 1 and 2” section.

#### Dietary intake and oral hygiene

Subjects and their parents completed a dietary intake and dental hygiene questionnaire created for this study. The questionnaire evaluated the presence and frequency of specific dietary consumptions and dental hygiene habits. Diet and medication items assessed included between meal snacks, carbonated beverages, swishing and holding drinks in the mouth, fruit juices before bed, energy drinks, medicines in a syrup form, chewing Vitamin C tablets, asthma inhalers, citrus fruits, yogurt, and pickles. Oral hygiene and habit items included grinding teeth at night, tooth brushing in the morning, tooth brushing before going to bed, use of fluoride treatments, receiving regular dental care, presence of fluoride in drinking water, type of toothbrush, and frequency of brushing. The dietary intake and oral hygiene questionnaire is located in the “Additional files 1 and 2” section.

#### Esophageal histology

An endoscopy with biopsies was performed as part of routine medical care in all subjects. A minimum of two biopsies were obtained from the lower one-third of the esophagus in all subjects and were utilized for histologic examination. All biopsy specimens were stained with Hematoxylin and Eosin stain and evaluated as part of routine care by a board certified pediatric pathologist with determination of eosinophil density reported as number of cells per high power field (hpf)(400X). The presence of no eosinophils was considered normal, 1–14 eosinophils/hpf as GER esophagitis, and ≥15 eosinophils/hpf as indicative of eosinophilic esophagitis. The results of standard biopsies taken from the lower third of the esophagus during an endoscopy were used to divide subjects into either the control group (Non-GERD) or the GERD group (i.e. those with histologic esophagitis). Subjects that had eosinophilic esophagitis confirmed on biopsy were eliminated from the study.

#### Dental examination

At the endoscopy appointment, a dental examination was performed by one of two licensed dentists to assess for the presence and severity of dental erosions (as described below), signs of attrition (bruxism), enamel hypoplasia or defects, and the stage of dentition (primary, mixed, and permanent). The dentists were calibrated and reliability was monitored throughout the study to insure intra- and interrater reliability was maintained at a rate of 80% or higher. Intra-oral photographs were standardized and taken on all patients to document the facial, lingual/palatal, and occlusal surfaces of the dentition only. The dental exam did not assess dental conditions other than those listed above. The parent or legal guardian was informed of any conditions that were found during the child’s dental exam and the child was referred to their personal dentist or another dentist in the community.

During the dental examination, dental erosions were classified according to their clinical appearance by using a modified index developed by O’Brien [18]. O’Brien used a partial recording system for measuring tooth erosion in children in an epidemiological survey, where only the facial and lingual surfaces of the primary and permanent maxillary incisor teeth were scored. In the current study the O’Brien index was utilized to score erosion on the facial, lingual and incisal/occlusal surfaces of all primary and permanent teeth. The scoring criteria of the O’Brien index assigns the scores 1, 2, and 3 to lesions into enamel, into dentin, or in close proximity to the pulp, respectively [18]. The lesion’s depth is always scored before the area covered by the lesion because the later criterion refers to the area of the worst depth observed [18]. For the assessment of lesion area, the O’Brien index assigns scores of 1, 2, and 3 to lesions involving one third, one third up to two thirds, or more than two thirds, respectively, to the tooth surface affected [18]. For criteria of both lesion depth and area, a score of 0 refers to sound tooth surface or no surface area involvement, respectively. A score of 9 is given for both lesion depth and area if an assessment cannot be made on the tooth [18].

### Statistics

Descriptive data (means, standard deviation [SD], frequencies and percentages) were computed for continuous and categorical variables on demographic, related symptoms, and oral behaviors, respectively. Baseline comparisons of the GERD and Non-GERD participants were analyzed using Mann–Whitney U test as distributions were largely skewed (on average ≥ 2.0) with occasional outliers. As the distribution of teeth with higher erosion scores were highly skewed in the two diagnostic groups by type of tooth (primary versus permanent), tooth-level erosion data were subsequently dichotomized for analysis to having any Erosion vs. No Erosion. Data were analyzed by fitting mixed effect logistic regression model to assess tooth-level erosion by diagnostic group clustered within individual participant as the random effect (STATA 11.0 SE, StataCorp LP, College Station, TX) to derive maximum-likelihood and variance estimates. As previous subgroup analyses showed that the effect of tooth type (primary versus permanent tooth) was significantly related to erosion scores, tooth type was included in the model to control for those effects at the tooth level. For this model, erosion at the tooth level (present/absent) was modeled as the outcome clustered within individual, with diagnostic group (GERD/Non-GERD) and tooth type (primary/permanent) as predictor variables All analyses were conducted at the α = .05.

## Results

Baseline characteristics (means ± standard deviation [SD]) and symptoms of the two groups are displayed in Table 1. All of the participants were pediatric gastrointestinal patients requiring biopsy for diagnosis of their chief complaint. Twenty-five subjects were consented and enrolled. Of those, six (27%) had biopsy confirmed diagnoses of GERD, 16 had normal esophageal histology (Non-GERD), and three were diagnosed with eosinophilic esophagitis. These three participants were excluded from analyses. The mean ages of the GERD and Non-GERD participants were equivalent at 12.5 years each. Characteristics related to symptoms, dietary habits and oral hygiene behaviors across both diagnostic groups were highly variable and positively skewed. Mann–Whitney U test was used to compare groups as the required assumptions for parametric analyses were not met. Results showed that the composite abdominal pain score was statistically significantly greater for the GERD group (U = 18.5, DF = 1, *p* = .029). Although there were some observable trends in the data, no other comparisons were determined to be statistically different.

**Table 1:** Characteristics of GERD vs. Non-GERD Subjects

Characteristics	Non-GERD Subjects *N* = 16	GERD Subjects *N* = 6	*p*
Mean Age in Years (SD)	12.5 (3.8)	12.5 (4.1)	NS
Abdominal Pain Composite Score (Frequency/Week X Severity)	12.9 (7.6)	28.8 (21.4)	.029
Fried Food Consumption/Week	5.5 (8.5)	4.2 (8.3)	NS
Spicy Food Consumption/Week	3.3 (6.0)	4.8 (9.7)	NS
Nausea/Week	4.4 (4.7)	6.6 (8.1)	NS
Vomiting/Week	1.7 (4.5)	1.0 (1.5)	NS
Heartburn/Week	1.8 (4.6)	1.0 (3.4)	NS
Burping/Week	1.4 (5.2)	0.7 (1.6)	NS
Poor Sleep/Week	2.5 (7.1)	4.7 (11.4)	NS
Brush AM/Week	3.9 (3.2)	5.0 (3.1)	NS
Brush PM/Week	3.7 (3.1)	3.8 (3.5)	NS
Night Bruxing/Week	0.6 (1.8)	2.5 (3.5)	NS
Snacking/Week	5.3 (3.1)	7.8 (5.0)	NS
Soda/Week	2.0 (2.0)	3.6 (2.1)	NS
Antacids/Week	1.9 (3.8)	0.9 (1.6)	NS

Within the 22 participants, 586 teeth were evaluated for both extent of erosion depth and erosion area per tooth (Tables 2 and 3). Each tooth was additionally scored as to whether it was a primary or permanent tooth. Of the 586 teeth, 438 were permanent teeth and 148 were primary teeth. Overall, the tooth-level prevalence of erosion in the GERD group was 22/153 (14.4%) compared to 84/438 (19.2%) in the Non-GERD group. A descriptive assessment of the distribution of tooth depth showed that permanent teeth were more likely to have scores of 0 (no erosion – depth) compared to primary teeth, irrespective of group. The proportion of permanent teeth in the Non-GERD and GERD groups without erosion was 90 and 91%, respectively. In contrast, 51 and 69% of primary teeth in the Non-GERD and GERD groups, respectively, had no observable erosion depth scores. A similar pattern was observed for permanent/primary teeth with respect to total tooth area.

**Table 2:** Distribution of erosion characteristics for groups and primary/permanent teeth: percent of teeth (95% CI) with tooth depth erosion scores

Group/Tooth Type	Tooth Depth Erosion Scores		
	0	1	2
Non-GERD Permanent Teeth (*N* = 324)	90.4 (87.3, 93.8)	8.3 (5.2, 11.4)	1.2 (.03, 2.5)
Non-GERD Primary Teeth (*N* = 109)	51.4 (42.2, 60.6)	42.2 (33.0, 51.5)	6.4 (2.8, 11.0)
GERD Permanent Teeth (*N* = 114)	91.2 (86.0, 95.6)	8.8 (4.4, 14.0)	0
GERD Primary Teeth (*N* = 39)	69.2 (53.8, 84.6)	20.5 (10.3, 33.3)	10.3 (2.6,20.5)

**Table 3:** Distribution of area erosion scores for groups and primary/permanent teeth: % of teeth (95% CI) with tooth area erosion scores

Group/Tooth Type	Tooth Area Erosion Scores			
	0	1	2	3 or greater
Non-GERD Permanent Teeth (*N* = 324)	90.4 (87.3, 93.8)	5.9 (3.4, 8.6)	1.2 (.03, 2.5)	2.5 (.09, 4.3)
Non-GERD Primary Teeth (*N* = 109)	51.4 (42.2, 60.6)	26.6 (18.3, 34.9)	13.8 (7.3, 21.1)	8.3 (3.7, 13.8)
GERD Permanent Teeth (*N* = 114)	91.2 (86.0, 95.6)	4.4 (0.9, 7.9)	0.9 (0, 3.5)	3.5 (0.9, 7.0)
GERD Primary Teeth (*N* = 39)	69.2 (53.8, 84.6)	5.1 (0, 12.8)	10.3 (2.6, 20.5)	15.4 (5.1, 28.2)

Initially, mixed effects ordinal logistic regression was used to model depth and area of erosion, separately, as a function of group while controlling for whether the tooth was primary or permanent. Because this analysis yielded highly unstable estimates for the higher categories of severity, depth and area were subsequently dichotomized into no erosion or any erosion, and mixed effect logistic regression computed. Results from the logistic mixed effects regression model for erosion are displayed in Table 4. This analysis showed that diagnostically confirmed GERD was not a significant predictor for erosion (*p* = 0.482). However, primary teeth had significantly greater odds of having erosion compared to permanent teeth (Odds Ratio (OR) = 3.64, *p* = .0001) irrespective of diagnostic group. In order to test whether there was a potential interaction between group and tooth type, an interaction term was added to the model and likelihood-ratio test performed. Addition of the interaction term did not improve model fit (LR Chi Square = 0.01, *p* = 0.923) and was dropped from the final model.

**Table 4:** Mixed effects logistic regression (*N* = 22 Subjects, 586 Teeth)

Outcome: Tooth Erosion Dichotomized			
Variable	OR	95% CI	p
Primary Tooth Type	3.64	2.57, 4.70	.0001
Group	−0.61	−2.31, 1.09	.482

## Discussion

The diagnosis of GERD may be complicated and difficult in pediatric patients who may be non-verbal and/or whose symptoms are not clearly discernible. Symptom criteria have been shown to be an unreliable predictor of GERD in children [1]. Commonly used diagnostic tests include intra-esophageal pH monitoring (pH study) and endoscopy with biopsy. A pH study involves continuous monitoring of esophageal pH to determine esophageal acid exposure and has established normal ranges. Endoscopy allows for evaluation of gross esophageal appearance and for esophageal biopsy to determine the presence of histologic inflammation from acid exposure. No tests have been shown to have high sensitivity and specificity for the diagnosis of GERD in children and adolescents. The ability of various tests to predict specific non-gastrointestinal complications remains to be determined [1].

There have been eight previous studies evaluating an association between GERD and dental erosions in children and adolescents [4, 10–16]. Subjects with GERD were compared to a control group in five of these studies [4, 12–14, 16]. The definition of GERD varied across these studies being defined by an abnormal pH study in two, abnormal endoscopy and/or histology in two, and predominantly by either clinical history or endoscopy in the other. Studies have also varied by whether they analyzed by subject or by tooth and by proportions of primary and permanent teeth evaluated. In the current study, we found dental erosions to be more prevalent in primary teeth. A limitation of this study was that the sample of subjects was not homogeneous, so the permanent teeth may have been subjected to the effect of the acid for different periods of time in the different subjects. Furthermore, the duration of the disease might have affected differently the teeth of each patient.

The two studies defining GERD by pH study parameters have had mixed results. Wild et al. evaluated only permanent teeth and found no difference in the percentage of subjects or the percentage of teeth with erosions comparing GERD subjects to controls [14]. Ersin et al. evaluated both primary and permanent teeth in a younger group and found erosions to be increased in both tooth types in subjects with GERD [13]. Erosions were present in 76% of GERD subjects as compared to 24% of controls. In both studies, pH studies were only performed in the GERD group and a different methodology was used to access tooth erosion.

The two studies defining GERD by endoscopy had somewhat mixed results. Dashan et al. defined GERD by gross endoscopic appearance or by histology, however the frequency of abnormal histology is not reported [12]. In the study by Dashan et al. many of the affected subjects had primary dentition [12]. They found that GERD subjects were much more likely to have dental erosions [12]. They also found that their subjects were much less likely to report brushing after every meal [12]. Linnett et al. compared subjects with histologic esophagitis to sibling controls who did not have histology assessed [4]. There were no differences in the percentage of subjects or the percentage of primary teeth with erosions in GERD subjects as compared to controls [4]. GERD subjects were more likely than controls to have erosions in permanent teeth (4% vs. 0.8%) [4]. The current study adds to the findings of Linnett et al. in that our study is the first to also define controls by histologic criteria (or by any diagnostic test). We found no differences in the percentage of erosions of either primary or permanent teeth between subjects with and without histologic esophagitis. It is possible that our controls contained subjects with GER without esophagitis. This will always be a challenge as symptoms have been shown not to be predictive of GERD in children. However, the overriding goal of the current study was to evaluate the ability of a single diagnostic test to predict dental erosions.

This study had some investigational limitations. The results are based upon a convenience sample of patients seeking care at a single medical institution and the majority of erupted teeth evaluated were permanent. While the findings are interesting, expanding the study to include more patients with primary dentitions would be beneficial.

## Conclusions

In conclusion, in this study dental erosions do not appear to be associated with histologically confirmed GERD. Future studies are needed to see if additional diagnostic tests, or combinations of tests, increase the ability to predict dental erosions.

## Additional files

Additional file 1: GERD Symptoms Questionnaire - The questionnaire assessed the presence of various symptoms and if present, the subjects rated severity on a 3-point Likert scale (mild, moderate, severe) and estimated episode frequency per week [17]. Symptoms included pain (in general and with consumption of fried and spicy foods), dysphagia, nausea, vomiting/regurgitation, chest pain, heartburn, excessive belching, bad breath, excessive crying, poor sleep, and relief from anti-acid medications. (DOC 62 kb)

Additional file 2: Food-Dental Care Questionnaire - The questionnaire evaluated the presence and frequency of specific dietary consumptions and dental hygiene habits. Diet and medication items assessed included between meal snacks, carbonated beverages, swishing and holding drinks in the mouth, fruit juices before bed, energy drinks, medicines in a syrup form, chewing Vitamin C tablets, asthma inhalers, citrus fruits, yogurt, and pickles. Oral hygiene and habit items included grinding teeth at night, tooth brushing in the morning, tooth brushing before going to bed, use of fluoride treatments, receiving regular dental care, presence of fluoride in drinking water, type of toothbrush, and frequency of brushing. (DOCX 23 kb)

## Abbreviations

GER: Gastroesophageal reflux
GERD: Gastroesophageal reflux disease
