# Supplementary material for: Is histologic esophagitis associated with dental erosion: a cross-sectional observational study?
Source: BMC Oral Health. 2017 Aug 10;17:116. doi: 10.1186/s12903-017-0408-z (PMC5553729; doi:10.1186/s12903-017-0408-z)
Supplement: Supplementary file 1 — GERD Symptoms Questionnaire - The questionnaire assessed the presence of various symptoms and if present, the subjects rated severity on a 3-point Likert scale (mild, moderate, severe) and estimated episode frequency per week [17]. Symptoms included pain (in general and with consumption of fried and spicy foods), dysphagia, nausea, vomiting/regurgitation, chest pain, heartburn, excessive belching, bad breath, excessive crying, poor sleep, and relief from anti-acid medications. (DOC 62 kb) [file 12903_2017_408_MOESM1_ESM.doc]

GERD SYMPTOM QUESTIONNAIRE

| **For the below questions please mark no or yes.**  **If yes, then grade severity and answer frequency per week.** | **No** | **Yes** | **Severity**  **(Circle One)** | **Frequency**  **per week** |
| --- | --- | --- | --- | --- |
| 1. Does your child experience abdominal (stomach) pain? |  |  | Mild / Moderate / Severe |  |
| 1. Does your child experience pain or indigestion from fried foods? |  |  | Mild / Moderate / Severe |  |
| 1. Does your child experience pain or indigestion from spicy foods? |  |  | Mild / Moderate / Severe |  |
| 1. Does your child have difficulty swallowing (dysphagia)? |  |  | Mild / Moderate / Severe |  |
| 1. Does your child experience nausea? |  |  | Mild / Moderate / Severe |  |
| 1. Does your child experience vomiting/regurgitation? |  |  | Mild / Moderate / Severe |  |
| 1. Does your child have frequent ear, nose or throat problems? |  |  | Mild / Moderate / Severe |  |
| 1. Does your child complain of chest pain? |  |  | Mild / Moderate / Severe |  |
| 1. Does your child complain of heartburn? |  |  | Mild / Moderate / Severe |  |
| 1. Does your child experience frequent respiratory problems? |  |  | Mild / Moderate / Severe |  |
| 1. Does your child have periods of excessive burping or belching? |  |  | Mild / Moderate / Severe |  |
| 1. Does your child have bad breath? |  |  | Mild / Moderate / Severe |  |
| 1. Does your child have periods of excessive crying or fussiness? |  |  | Mild / Moderate / Severe |  |
| 1. Does your child experience poor sleep? |  |  | Mild / Moderate / Severe |  |
| 1. Does your child get relief when they take anti-acids (i.e. omeprazole (Prilosec®, Dexilant®), lansoprazole (Prevacid®), famotidine (Pepcid®), esomeprazole (Nexium®), ranitidine (Zantac®) Maalox®, Gaviscon®, Rolaids®, Tums®, or Alka-Seltzer®) ? |  |  | **Relief from Anti-Acids**  **(circle one)**  Mild Relief  Moderate Relief  Total Relief | Frequency the child takes anti-acids per week? |

Patient or Legal Guardian Signature __________________________________________ Date _______________

Print Name ______________________________________________________________
