# Supplementary material for: Is histologic esophagitis associated with dental erosion: a cross-sectional observational study?
Source: BMC Oral Health. 2017 Aug 10;17:116. doi: 10.1186/s12903-017-0408-z (PMC5553729; doi:10.1186/s12903-017-0408-z)
Supplement: Supplementary file 2 — Food-Dental Care Questionnaire - The questionnaire evaluated the presence and frequency of specific dietary consumptions and dental hygiene habits. Diet and medication items assessed included between meal snacks, carbonated beverages, swishing and holding drinks in the mouth, fruit juices before bed, energy drinks, medicines in a syrup form, chewing Vitamin C tablets, asthma inhalers, citrus fruits, yogurt, and pickles. Oral hygiene and habit items included grinding teeth at night, tooth brushing in the morning, tooth brushing before going to bed, use of fluoride treatments, receiving regular dental care, presence of fluoride in drinking water, type of toothbrush, and frequency of brushing. (DOCX 23 kb) [file 12903_2017_408_MOESM2_ESM.docx]

|  | Yes | No | Frequency per Week? |
| --- | --- | --- | --- |
| 1. Does your child snack between their 3 main meals? |  |  |  |
| 1. Does your child drink carbonated beverages (soda, pop)? |  |  |  |
| 1. Does your child have a habit of swishing or holding their drink in their mouth for long periods of time? |  |  |  |
| 1. Does your child drink fruit juice before bed? |  |  |  |
| 1. Does your child drink energy drinks? |  |  |  |
| 1. Does your child take medicine that is in the form of syrup daily? |  |  |  |
| 1. Does your child chew Vitamin C tablets? |  |  |  |
| 1. Does your child complain of a dry mouth? |  |  |  |
| 1. If your child has asthma, do they use an inhaler? |  |  |  |
| 1. Does your child eat citrus fruits (i.e. orange, tangerine, tangelo, orange, lime, lemon, kumquats, grapefruit, clementine, pummelo)? |  |  |  |
| 1. Does your child eat yogurt? |  |  |  |
| 1. Does your child eat pickles? |  |  |  |
| 1. Does your child grind their teeth at night? |  |  |  |
| 1. Does your child brush their teeth in the morning? |  |  |  |
| 1. Does your child brush their teeth before they go to bed? |  |  |  |
|  | Yes | No | Frequency per Week? |
| 1. Does your child use fluoride treatments at home? |  |  |  |
| 1. Does your child receive regular dental care? |  |  |  |
| 1. Is their fluoride in your child’s drinking water? |  |  |  |
| 1. What type of toothbrush does your child use? **Circle one:** | Soft, Medium, Hard, Electric | | |
| 1. How often does your child brush their teeth a day? |  | | |
